# Resting-state functional connectivity related to empathy and sensory processing sensitivity

**DOI:** 10.3389/fnhum.2026.1940950

**Published:** 2026-09-03

**Authors:** Amanda M. McQuarrie, Lorna S. Jakobson, Stephen D. Smith

**Affiliations:** 1 Department of Psychology, University of Manitoba, Winnipeg, MB, Canada; 2 Department of Psychology, The University of Winnipeg, Winnipeg, MB, Canada

**Keywords:** empathy, functional connectivity (FC), personal distress, resting-state magnetic resonance imaging, sensory processing sensitivity (SPS)

## Abstract

**Introduction:**

Sensory processing sensitivity (SPS) is a personality trait that has both positive features (SPS+) such as sensitivity to subtle cues, and negative features (SPS−) such as sensitivity to unpleasant stimuli or situations. Both SPS feature clusters are related to empathy. This study examined the neural correlates of SPS as it relates to empathy in the brain at rest.

**Methods:**

In a sample of university students (*N* = 172), we identified links between SPS facets and multiple aspects of empathy measured via self-report. Subsequently, resting-state functional magnetic resonance imaging scans from 40 of these participants (M_age_ = 21.10 years; 22 females) were analyzed, using neural regions associated with empathy as seeds. Second-level correlation analyses identified associations between functional connectivity (FC) for each seed-to-voxel connection and each facet of SPS.

**Results:**

SPS+ was associated with increased FC between medial prefrontal cortex (mPFC) and left superior parietal lobule, right intracalcarine cortex, and bilateral lateral occipital cortex (LOC). SPS− was associated with increased FC between the left inferior frontal gyrus and right frontal pole; right insula and primary somatosensory cortex; mPFC and left LOC; and precuneus and left frontal pole. SPS− was also associated with decreased FC between the left inferior frontal gyrus and left LOC; left insula and brainstem; precuneus and left occipital pole; and precuneus and right cerebellum.

**Conclusion:**

These patterns may relate to specific features of SPS including enhanced visual processing, deeper processing of information, and heightened interoceptive and emotional processing, which may contribute to the strong feelings of empathy reported by individuals with SPS, and to emotion dysregulation that may exacerbate feelings of personal distress in those possessing strong SPS− traits when witnessing others’ suffering.

## Introduction

The personality trait of sensory processing sensitivity (SPS) is characterized by heightened sensitivity to the internal and external environment, strong emotional reactivity, and processing stimuli at a deeper level^1^ (Aron and Aron, 1997; Aron et al., 2012). SPS can be found in approximately 30% of the general population (Lionetti et al., 2018). The overarching trait can be divided into positive and negative components (Attary and Ghazizadeh, 2021; De Gucht et al., 2022; Jakobson et al., 2024). The positive component of SPS (SPS+) encompasses one’s sensitivity to interpersonal cues, aesthetic qualities of the environment, and subtle stimuli (e.g., scents and textures), as well as one’s awareness of how these cues affect one at an emotional level (e.g., how the weather affects one’s mood). The negative SPS trait cluster (SPS−) includes sensitivity to unpleasant stimuli (e.g., loud noises) and being prone to overstimulation.

The two clusters of SPS traits show distinctly different relationships to other personality traits and to aspects of affective processing. For example, SPS+ traits are linked to the Big Five personality traits of openness and conscientiousness (Attary and Ghazizadeh, 2021), the capacity to attend to body sensations and connect them to emotional states (Jakobson et al., 2026), and adaptive forms of self-regulation such as cognitive appraisal (Eşkisu et al., 2022). In contrast, the SPS− trait cluster is associated with alexithymia, neuroticism, shyness, anxiety, and depression (Attary and Ghazizadeh, 2021; Jakobson et al., 2024) as well as maladaptive forms of emotion regulation such as emotion suppression (Eşkisu et al., 2022). The purpose of the present investigation was to examine links between these two facets of SPS and another domain of affective processing, namely empathy.

Empathy is a multidimensional human capacity. It is defined as the process of identifying and feeling the same emotion as another person, while recognizing that the emotion belongs to the empathy target (Cuff et al., 2016; Håkansson Eklund and Summer Meranius, 2021; McQuarrie et al., 2023, 2025). Research into individual differences in empathy frequently rely on self-report measures to gather information about different tendencies related to this overarching construct. For example, the Empathy Index (Jordan et al., 2016) measures the tendency to mirror another person’s observable emotions and behavior (i.e., to exhibit emotional and behavioural contagion) and the Interpersonal Reactivity Index (Davis, 1983) measures the tendencies to feel concern for those experiencing difficulties, adopt others’ psychological points of view, and become absorbed in narrative works and fantasize or mentally transport oneself into the lives of the characters depicted. A fourth subscale of this latter measure taps into the extent to which one feels anxious or uneasy in tense settings. This self-oriented measure is important for empathy researchers to consider, as being overwhelmed by feelings of *personal* distress may interfere with one’s ability to regulate one’s own behavioural responses in ways that are sensitive to others (Jordan et al., 2016).

Both the SPS+ and SPS− components have been positively linked to emotional contagion, perspective taking, empathic concern, and fantasizing (McQuarrie et al., 2025). However, the two trait clusters differ with regard to their links to the experience of personal distress while empathizing, with SPS+ traits predicting lower levels of distress and SPS− traits predicting higher levels (McQuarrie et al., 2025). It may be that individuals who express high levels of SPS are generally better able to empathize with others because they are more sensitive to salient contextual, affective, and body-based cues, and because they process thoughts and feelings more deeply than those who express low levels of SPS (Aron et al., 2012; Jakobson et al., 2024; McQuarrie et al., 2023). However, we propose that their ability to regulate negative empathy-induced emotions may depend, directly or indirectly, on how strongly they express SPS+ and SPS− traits. Those scoring high on SPS+ traits appear to show greater resilience to affective distress arising from exposure to others’ trauma via their enhanced ability to stay in the present moment (Gulla and Golonka, 2021) and to experience fulfillment, joy, and meaning from helping others (McQuarrie and Jakobson, 2025). In contrast, the “empathic overarousal” (Hoffman, 2008), problems with emotion regulation (Brindle et al., 2015), and low levels of self-compassion and compassion satisfaction (McQuarrie and Jakobson, 2025) seen in those scoring high on SPS− traits may increase the risk of experiencing empathic distress—and negative affectivity more generally.

Despite evidence of strong links between facets of SPS and empathy, there has been relatively little research examining the neural substrates of these associations. One neuroimaging technique that may be particularly useful in this regard is resting-state functional magnetic resonance imaging (rsMRI), a measure of neural activity that occurs while an individual is not performing a cognitive or motoric task. rsMRI measures functional connectivity (FC), a value representing the degree of correlated neural activity between two or more topographically disparate regions of the brain (Biswal et al., 1995; Lee et al., 2013). Using this technique, Acevedo et al. (2021) found that SPS as an overarching trait was associated with increased FC in regions involved with attention (e.g., middle frontal gyrus and anterior cingulate cortex) and memory (e.g., precuneus and hippocampus). Associations between SPS and reduced limbic system connectivity (e.g., between the amygdala, periaqueductal gray and insula) were also identified (Acevedo et al., 2021). Another study investigating associations between FC, SPS, and attention deficits identified increased FC between the rostral hippocampus and the inferior parietal lobule (Zhou et al., 2025). SPS was found to mediate the relationship between the FC of these two regions and attention deficits. The authors hypothesized that the increased FC between the hippocampus and inferior parietal lobule may explain attention deficits related to SPS through heightened processing of sensory stimuli, which could potentially lead to overstimulation (Zhou et al., 2025). These two studies provide some evidence of the neural underpinnings of SPS as a whole trait, although the specific neural connections between SPS (in particular, the SPS− and SPS+ components) and empathy have not been yet explored.

In the current study, neural regions commonly involved in empathy were used as seeds when analyzing rsMRI data. Meta-analyses of neuroimaging studies involving empathy-specific tasks have identified the midcingulate/anterior cingulate cortex and anterior insula as forming a “core” empathy network (Fan et al., 2011; Timmers et al., 2018; Jauniaux et al., 2019). Other neural regions commonly associated with empathy tasks include the inferior frontal gyrus (IFG), medial prefrontal cortex (mPFC), precuneus, and temporoparietal junction (Timmers et al., 2018; Jauniaux et al., 2019; Kogler et al., 2020). Empathy is also reflected in the resting brain, particularly with connectivity between the mPFC and posterior cingulate cortex, insula, precuneus, and anterior cingulate cortex (Li et al., 2014; Takeuchi et al., 2014; Kim et al., 2017; Tremblay et al., 2022).

Specific objectives of the current study included: (1) to replicate prior findings supporting the relationship between SPS and self-reported empathy; (2) to identify FC associated with SPS+ between the seeds mentioned above and the rest of the brain; and (3) to measure FC associated with SPS− between the seeds and the rest of the brain. Given the literature reviewed above suggesting strong links between both components of SPS and empathy, it was hypothesized that strong expression of these trait clusters would be associated with alterations in FC between the empathy-related seeds and other regions. However, we also expected that SPS− traits, in particular, would be more strongly associated with connectivity in areas involved in processing emotionally salient cues, self-referential processing, and emotion regulation.

## Methods

### Participants

There were two phases to this experiment. Initially, 186 adult participants were recruited from an undergraduate psychology class for phase I. After cleaning (described below), the final sample included 172 participants (102 men, 67 women, 2 transgender men, 1 non-binary individual; M_age_ = 21.85 years, SD_age_ = 5.39 years). Data obtained from these participants were used to assess the reliability of the self-report measures and to address our first study objective. An *a priori* power analysis conducted using G*Power 3.1.9.7 (Faul et al., 2009) confirmed that the sample size provided more than adequate power (> 0.80) to detect medium effects in the multiple regressions planned for this phase of the research.

Based on eligibility criteria and availability, 41 participants (19 men, 22 women; age range 18–34) were then recruited from this sample to complete an MRI scanning session in phase II. Exclusion criteria included: a self-reported history of neurological disorders or significant head injury (to reduce potential confounding variables); not having normal or corrected-to-normal vision (relevant for a task-based scan not included in the present study); an inability to understand instructions presented in English; and non-Canadian citizenship. The latter restriction was put in place in an attempt to minimize potential confounds linked to cultural or related factors on processes associated with empathy while not inadvertently excluding specific ethnic or racial minority groups disproportionately. We acknowledge, however, that such factors would be impossible to control for completely, given that Canadian society is highly multicultural. Inclusion criteria included completing and passing an MRI screening questionnaire.

Participants received credit toward a research participation option for taking part in this study. Individuals who took part in the scanning session also received $20 CDN in monetary compensation to cover travel costs to and from the scanning location, and a complimentary copy of their structural brain scan.

### General procedure

Ethical approval was received from the university research ethics boards. Participants provided informed consent prior to participation. In phase I, participants completed self-report measures of SPS, empathy, and an MRI screening questionnaire (as well as several additional self-report measures used in a separate investigation) online via the Qualtrics survey platform (RRID: SCR_016728). A subset of the participants who were interested in completing the MRI scanning session and were eligible to do so (as determined by the MRI screening questionnaire) were contacted for phase II to book their session. Participants completed a 5-min structural scan and then an 11-min resting-state functional scan, where they were asked to lie completely still, focus on a fixation cross, and stay awake.

### Scanning parameters

MRI scans were conducted using a 3 Tesla Siemens Verio MRI scanner (Siemens Healthineers, Erlangen, Germany). Three-dimensional high-resolution T1-weighted gradient-echo anatomical MRI structural scans were used with a magnetization-prepared rapid gradient-echo (MP-RAGE) sequence. Structural scans used the following parameters: slices of 1-mm thickness, 0 gap, TR/TE = 1900/2.2 ms, in-plane resolution 0.94 × 0.94 mm, 256 × 256 matrix, with a field of view of 24 cm.

A whole-brain echo-planar imaging sequence was used to acquire functional data. Functional scans included the following parameters: slice thickness of 3-mm, 0 gap, TR/TE = 3000/30 msec, flip angle 90°, 64 × 64 matrix, also with a field of view of 24 cm.

### Personality questionnaires

#### Measures of SPS

The overarching concept of SPS was captured using two measures based on the recommendation by Aron et al. (2012): the Highly Sensitive Person Scale (HSPS; Aron and Aron, 1997) and the Orienting Sensitivity (OS) subscale of the Adult Temperament Questionnaire (Evans and Rothbart, 2007). The HSPS contains 27 items rated on a seven-option Likert scale from 1 (*not at all*) to 7 (*extremely*). The HSPS has three subscales: ease of excitation, which contains 12 items measuring the extent to which one becomes over-aroused by external stimuli; low sensory threshold, containing six items measuring discomfort related to sensory stimulation; and aesthetic sensitivity, with seven items measuring sensitivity to subtle stimuli and the aesthetic environment. HSPS subscale and total scores are found by calculating the mean of relevant items.

The OS adds to the HSPS by addressing sensitivity to subtle stimuli, and the richness of an individual’s inner life (Evans and Rothbart, 2007; Aron et al., 2012). The OS includes three five-item subscales: affective perceptual sensitivity, a measure of the emotional impact of low intensity stimuli; neutral perceptual sensitivity, measuring general awareness of low intensity internal and external stimuli; and associative sensitivity, measuring spontaneous cognitive processes such as imagery, intuition, and dreaming. Each of the 15 items is rated on a seven-point Likert scale, from 1 (*extremely untrue*) to 7 (*extremely true*), as well as an X (*not applicable*) option. Subscale scores are calculated by finding the mean of relevant items.

In the current study, the mean of the aesthetic sensitivity subscale of the HSPS and the three OS subscales was calculated to produce an SPS+ composite score, and the mean of the low sensory threshold and ease of excitation subscales of the HSPS served as an SPS− composite score (Jakobson et al., 2024). The Cronbach’s alpha was 0.83 for the SPS+ composite score and 0.89 for the SPS− composite, indicating good internal consistency.

#### Measures of empathy

Empathy and its related concepts were measured using the combined Interpersonal Reactivity Index (Davis, 1983) and Empathy Index (Jordan et al., 2016). Together, these two measures include 42 items with a five-option Likert scale from 0 (*does not described me well*) to 4 (*describes me very well*). The four subscales from the Interpersonal Reactivity Index include: Perspective Taking, adopting the viewpoint of another person; Personal Distress, the level of personal feelings of distress experienced when in tense interpersonal situations; Empathic Concern, feeling concern or sympathy for another; and Fantasy, adopting the feelings and actions of fictitious characters. The two additional subscales comprising the Empathy Index include: Behavioural Contagion, awareness of the extent to which one mimics the behaviours of those around them; and Empathy, awareness of emotional contagion, or the extent to which observing another’s emotions triggers similar feelings in oneself. Each subscale includes seven items, with responses summed to produce the subscale score. In the current sample, reliability of five of the six subscales was acceptable (𝛼 ≥ 0.72); however, the Behavioural Contagion subscale had an unacceptable reliability coefficient of 𝛼 = 0.54 (Tavakol and Dennick, 2011). As such, the scores on this subscale were not included in the analyses.

### Analyses

#### Personality questionnaires

To address our first objective, we assessed relationships between the two facets of SPS and self-reported empathy in the full sample (*N* = 172). This step was important for replication purposes but also to justify the use of the SPS composite scores as covariates in the rsMRI analyses and to aid in the interpretation of those analyses. The data were first cleaned by checking for duplicates (retaining the first completion for duplicate entries), outliers, and abnormal participant response patterns. Next, Little’s Missing Completely at Random test was run to determine if data were missing at random and, as this was the case, an expectation–maximization algorithm was used to estimate missing values. Following this, the subscale or composite scores for the self-report measures were computed and the distributions of these scores were checked for normality. Multiple linear regressions were run with the SPS trait cluster scores entered as predictors and the empathy subscales entered as outcome variables. Individual coefficients were estimated using 95% percentile confidence intervals estimated based on 1,000 bootstrap samples. Statistical analyses of the self-report measures were conducted using IBM SPSS Statistics for Microsoft, Version 28.

#### Neuroimaging analyses

For the neuroimaging data, the structural and functional neuroimaging files were converted into NifTI files through the NifTI creator function of SPM version 12.^2^ This allowed the rsMRI data to be analysed using the CONN functional connectivity toolbox (Whitfield-Gabrieli and Nieto-Castanon, 2012). Structural MRI data were centred to the coordinates (0,0,0), segmented into white matter, gray matter, and cerebrospinal fluid, and normalized to a standard brain atlas with Montreal Neurological Institute coordinates. Artifacts from head motion were regressed out (three translation and three rotation parameters). Functional data were also centred, segmented into white matter, gray matter, and cerebrospinal fluid, and normalized to a standard brain atlas. These data also underwent slice time correction and spatial smoothing utilizing an 8 mm Gaussian filter. Again, artifact interference was removed and denoising was performed utilizing the default 0.008 to 0.09 Hz bandpass filter. The structural and functional scans for each participant were co-registered.

First-level whole-brain analyses were run to examine the strength of the FC between seven key seeds (i.e., anterior cingulate cortex, insula, IFG, mPFC, precuneus, posterior cingulate cortex, and amygdala) and the rest of the brain. Second-level correlation analyses were then conducted with the SPS composite scores entered individually as covariates of interest. The associations between FC of each seed-to-voxel connection and the SPS+ and SPS− scores were measured first with all seeds entered into the general linear model as an initial, general test of significance. Post-hoc analyses then investigated the associations between an SPS trait cluster and connectivity of each seed individually. False discovery rate-controlling procedures were used to minimize the likelihood of false positives, with a cluster-threshold *p* < 0.05. Voxel threshold was set at *p* < 0.001 (uncorrected).

## Results

### Self-report data

Descriptive statistics of the self-report measures for the full sample (*N* = 172) are presented in Table 1. The results of the regression analyses run on these data (see Table 2) replicated many of the findings of McQuarrie et al. (2025). More specifically, as predicted: (a) participants reporting stronger SPS+ also reported stronger perspective-taking, feelings of concern, and ability to feel with fictitious characters, and weaker feelings of personal distress in response to witnessing others’ suffering; and (b) participants scoring higher on SPS− reported higher levels of awareness of emotional contagion and experienced more personal distress when empathizing with others. The fact that one or both SPS trait clusters significantly predicted each of the five self-report empathy measures justified our use of the SPS composite scores as covariates in the rsMRI analyses.

**Table 1:** Descriptive statistics for sensory processing sensitivity and empathy and related constructs.

Descriptive statistics	SPS+	SPS−	PT	EC	FS	EMP	PD
Mean/Sum	4.75	3.80	17.72	19.41	16.58	11.93	10.35
SD	0.77	1.06	4.98	4.45	5.39	4.87	5.02
Minimum	3.00	1.00	4.00	9.00	4.00	0.00	0.00
Maximum	6.70	6.38	28	28.00	27.00	23.00	22.00

Statistics calculated from the full sample (*N* = 172). SPS+, positive trait cluster of sensory processing sensitivity; SPS−, negative trait cluster of sensory processing sensitivity. Subscales of the Interpersonal Reactivity Index and Empathy Index: PT, perspective-taking; EC, empathic concern; FS, fantasy; EMP, empathy; PD, personal distress. SD, standard deviation.

**Table 2:** Regression analyses showing relationships between sensory processing sensitivity and empathy subscales.

Dependent variable	Predictor	*B*	Standard error	Bootstrapped 95% confidence interval		*p*-value (2-tailed)
				Lower	Upper	
PT	**SPS+**	**3.16**	**0.52**	**2.12**	**4.19**	**< 0.001**
	SPS−	−0.81	0.38	−1.51	0.03	0.04
EC	**SPS+**	**2.31**	**0.44**	**1.45**	**3.15**	**< 0.001**
	SPS−	0.44	0.37	−0.29	1.11	0.25
FS	**SPS+**	**2.67**	**0.55**	**1.49**	**3.66**	**< 0.001**
	SPS−	0.93	0.38	0.22	1.71	0.02
EMP	SPS+	1.20	0.47	0.27	2.14	0.02
	**SPS**−	**2.07**	**0.34**	**1.44**	**2.73**	**< 0.001**
PD	**SPS+**	**−2.10**	**0.38**	**−2.81**	**−1.31**	**< 0.001**
	**SPS**−	**3.09**	**0.32**	**2.45**	**3.70**	**< 0.001**

Regression analyses calculated from the full sample (*N* = 172). *B*, unstandardized coefficient; values shown in bold are significant at *p* < 0.01. 95% percentile confidence intervals were calculated using 1,000 bootstrap samples. SPS+ and SPS−, positive and negative trait clusters associated with sensory processing sensitivity. Subscales of the Interpersonal Reactivity Scale and Empathy Scale: PT, perspective-taking; EC, empathic concern; FS, fantasy; EMP, empathy; PD, personal distress.

### Functional resting-state analyses

The scan from one participant was removed due to image distortion. Thus, resting-state scans from 40 participants were analysed (18 men, 22 women; *M_age_ =* 21.10 years, *SD_age_* = 4.09 years), exceeding the average sample size of highly-cited fMRI studies published in recent years (Szucs and Ioannidis, 2020). Examination of descriptive statistics confirmed that the mean scores for the SPS composite and IRI/EI subscale scores for this subgroup of individuals were comparable to those seen in the full sample of individuals who had participated in phase I; as well, the scores on these variables for the scanned participants covered the full range of scores seen in the full sample. These observations suggest that the group who underwent imaging was representative of the larger sample.

**Figure 1:**
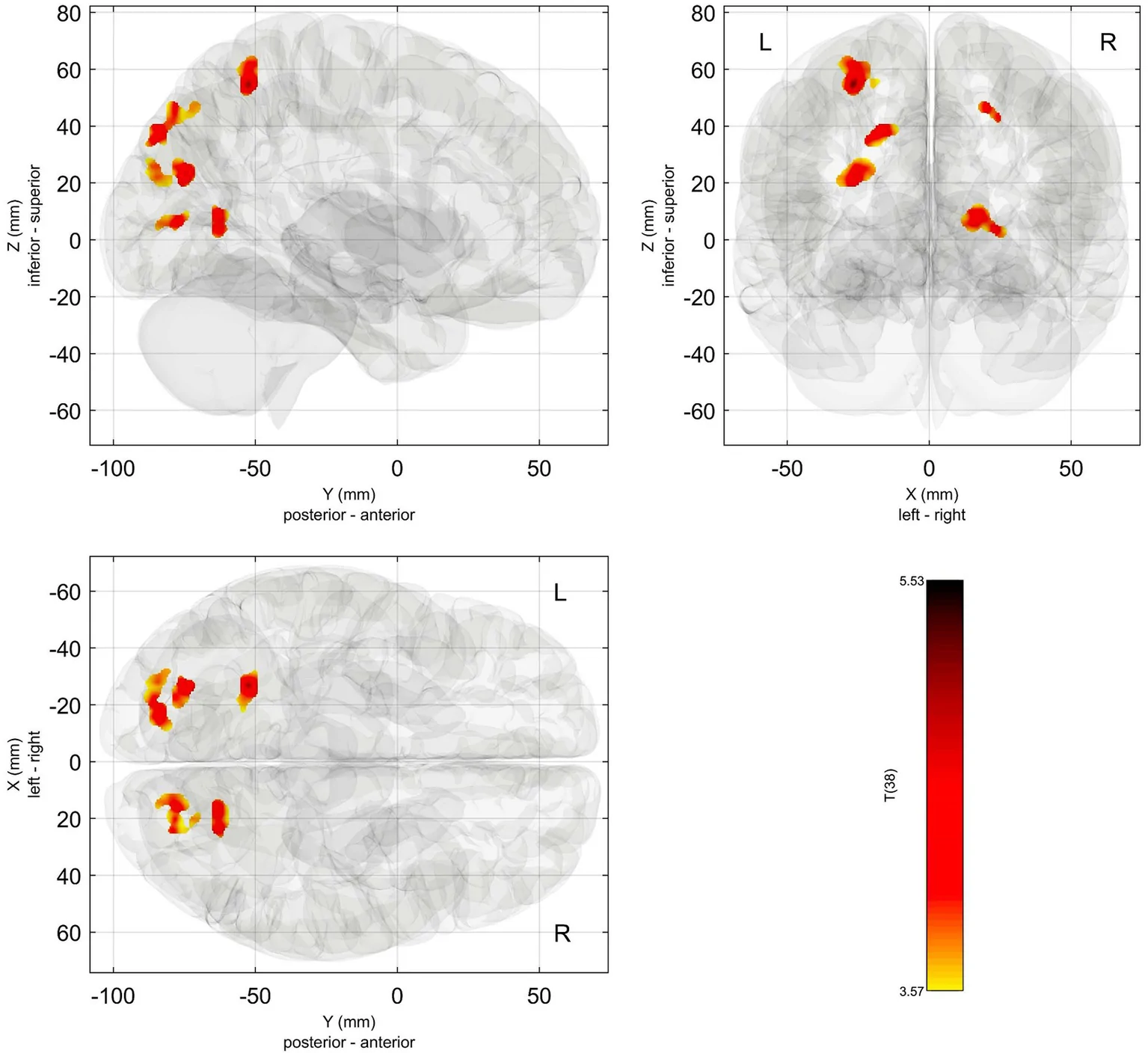
The positive SPS trait cluster score was correlated with functional connectivity between the medial prefrontal cortex and the highlighted regions, which include the left superior parietal lobule, right intracalcarine cortex (two separate clusters), left superior lateral occipital cortex (three separate clusters), and right superior lateral occipital cortex.

With the SPS+ trait score entered as a covariate, an omnibus *F*-test was first conducted including all seed regions. The general linear model produced positively correlated activity with a cluster located in the posterior inferior temporal gyrus (27 voxels, *p* = 0.03). Post-hoc analyses showed the mPFC as the only seed that produced significantly correlated connectivity. SPS+ scores were positively associated with FC between the mPFC and a cluster in the left superior parietal lobule and two separate clusters in the right intracalcarine cortex. Significant positive associations were also identified with FC between the mPFC and three separate but adjacent clusters in the left superior lateral occipital cortex (LOC): one extending into the cuneal cortex; one extending to the precuneus; and one solely within the left superior LOC. A final cluster within the right superior LOC was also significant. These associations are shown in Table 3 and Figure 1.

**Table 3:** Brain regions showing correlated connectivity with the positive sensory processing sensitivity trait cluster.

Brain region (hemisphere)	Cluster size	*p*-value (FDR)	Peak coordinates		
			*x*	*y*	*z*
Seed: mPFC					
Superior parietal lobule (L)	66	0.001	−28	−52	+54
LOC, superior division (L) Cuneal cortex (L)	43	0.009	−26	−74	+24
Intracalcarine cortex (R) Lingual gyrus (R) Precuneus	35	0.018	+18	−62	+08
LOC, superior division (L) Precuneus	32	0.021	−16	−84	+38
LOC, superior division (L)	27	0.030	−28	−84	+22
Intracalcarine cortex (R)	27	0.030	+16	−78	+06
LOC, superior division (R)	23	0.049	+20	−78	+46

All correlations were in the positive direction. FDR, false discovery rate corrected *p*-value. mPFC, medial prefrontal cortex. LOC, lateral occipital cortex. L, left; R, right.

**Figure 2:**
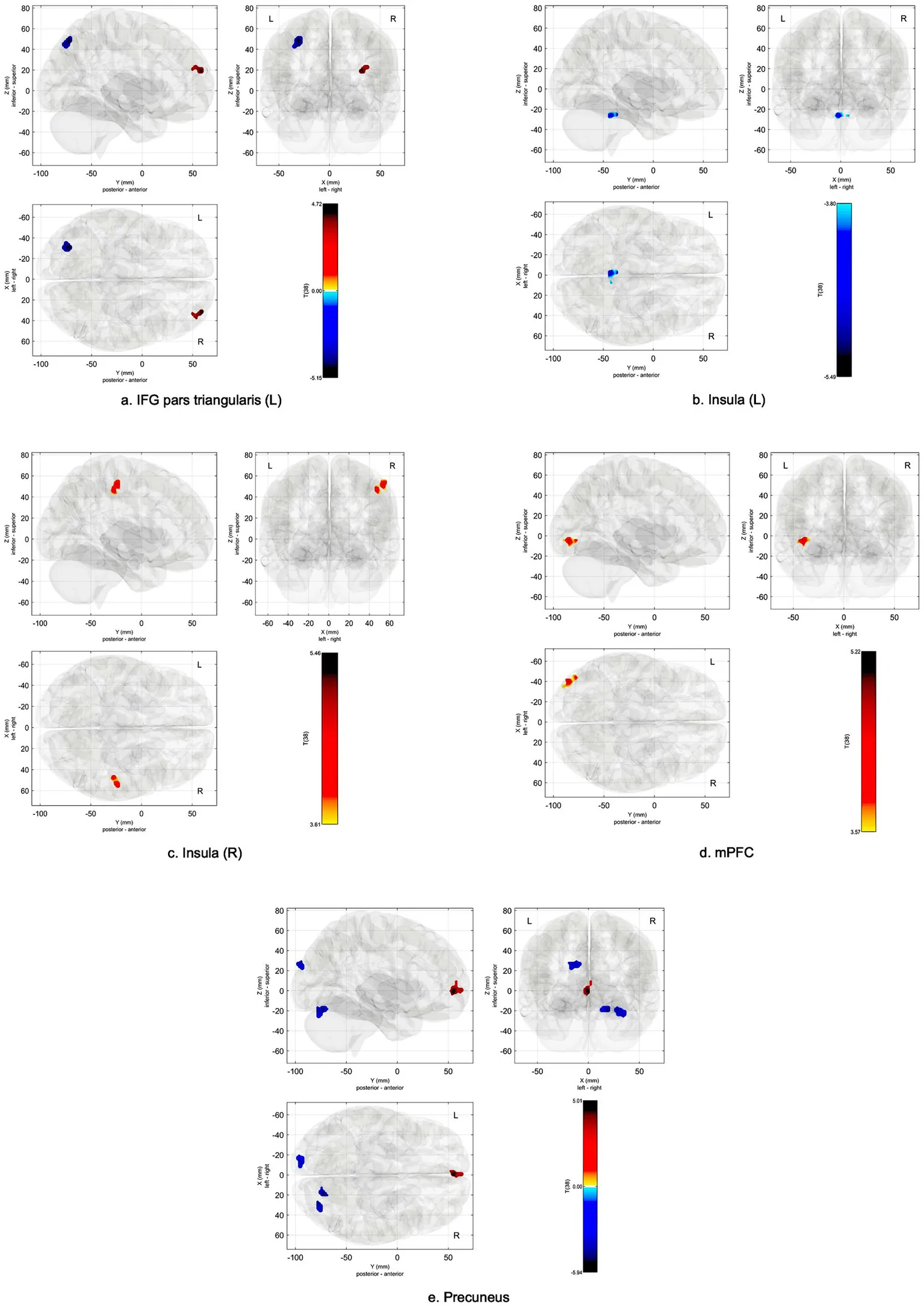
The negative SPS trait cluster score was correlated with functional connectivity between: **(A)** the left inferior frontal gyrus (IFG) pars triangularis and right frontal pole and left superior lateral occipital cortex; **(B)** left insula and brainstem; **(C)** right insula and right primary somatosensory cortex; **(D)** medial prefrontal cortex and left inferior lateral occipital cortex; and **(E)** precuneus and left occipital pole, right cerebellum Crus I, right cerebellum lobule VI, and left frontal pole.

The omnibus *F*-test with the SPS− trait score entered as the covariate showed positively correlated connectivity in the left superior LOC (two clusters: 59 voxels, *p* = 0.0002; and 22 voxels, *p* = 0.02), right frontal pole (48 voxels, *p* = 0.0005), precuneus (45 voxels, *p* = 0.0006), right cerebellum Crus I extending into lobule VI (26 voxels, *p* = 0.01), and right posterior inferior temporal gyrus (18 voxels, *p* = 0.05). Post-hoc analyses investigating each seed individually produced significant associations between this trait cluster and connectivity with multiple seeds. Connectivity positively associated with SPS− scores included: between the left IFG pars triangularis and the right frontal pole; between the right insula and the right primary somatosensory cortex extending into the anterior division of the supramarginal gyrus; between the mPFC and the left inferior LOC; and between the precuneus and the left frontal pole extending into the left paracingulate gyrus and right frontal pole (see Table 4 and Figure 2).

**Table 4:** Brain regions showing correlated connectivity with the negative sensory processing sensitivity trait cluster.

Brain region (hemisphere)	Correlation	Cluster size	*p*-value (FDR)	Peak coordinates		
				*x*	*y*	*z*
Seed: IFG, pars triangularis (L)						
LOC, superior division (L)	−	52	0.004	−32	−72	+48
Frontal pole (R)	+	29	0.050	+32	+58	+20
Seed: Insula (L)						
Brainstem	−	37	0.036	−02	−42	−26
Seed: Insula (R)						
Primary somatosensory cortex (R), Supramarginal gyrus, anterior division (R)	+	43	0.015	+54	−24	+52
Seed: mPFC						
LOC, inferior division (L)	+	37	0.036	−40	−84	−04
Seed: Precuneus						
Occipital pole (L)	−	46	0.007	−12	−94	+26
Cerebellum Crus I (R) Cerebellum VI (R) Fusiform gyrus (R)	−	43	0.007	+28	−78	−18
Cerebellum VI (R) Fusiform gyrus (R)	−	29	0.033	+18	−72	−18
Frontal pole (L) Paracingulate gyrus (L) Frontal pole (R)	+	41	0.007	−02	+56	+00

FDR, false discovery rate corrected *p*-value. IFG, inferior frontal gyrus. mPFC, medial prefrontal cortex. LOC, lateral occipital cortex. L, left; R, right.

FC negatively associated with SPS− scores included: between the left IFG pars triangularis and the left superior LOC; between the left insula and the brainstem; between the precuneus and the left occipital pole; between the precuneus and the right cerebellum Crus I extending into lobule VI and fusiform gyrus; and a separate cluster between the precuneus and right cerebellum lobule VI extending into the fusiform gyrus (see Table 4 and Figure 2).

In our sample no associations were found between SPS and altered connectivity between the three remaining key seeds (the anterior cingulate cortex, posterior cingulate cortex, and amygdala) and the rest of the brain.

## Discussion

In the current study we investigated correlations between SPS+ and SPS− traits and resting-state FC, with a focus on seeds previously identified as being involved in empathic processing. As this study used correlational analyses, causal assumptions cannot be made. Therefore, the possible explanations regarding the functions of neural regions provided below are considered speculative, although they are informed by prior research (Poldrack, 2006).

The focus on empathic processing was motivated by previous research findings regarding relationships between SPS and self-reported empathy (McQuarrie et al., 2025). The majority of these results were replicated in the current study. Specifically, reporting stronger SPS+ traits predicted scoring higher on perspective-taking, empathic concern, and fantasy, and lower on personal distress; and reporting stronger SPS− traits predicted scoring higher on emotional contagion and personal distress. These results support the view that individuals with SPS perceive themselves to be highly empathic, but that the degree to which they experience personal distress while empathizing varies as a function of how strongly they express SPS+ and SPS− traits, which are linked to lower and higher risk of personal distress, respectively.

In general, the functional connectivity results support and extend the findings of Acevedo et al. (2021). We will now discuss these results as they relate to several key features of SPS: enhanced sensory processing (specifically visual), deeper processing, heightened interoceptive and emotional processing, and problems with emotion regulation.

### Enhanced visual processing and deeper processing

The mPFC is involved in a number of higher-level cognitive functions, including self- and other-related processing, empathy/social cognition, emotion regulation, and decision-making (Etkin et al., 2011; Denny et al., 2012; Lieberman et al., 2019). The variety of functions is due in part to the numerous connections between the mPFC and other regions such as the anterior cingulate cortex, insula, temporoparietal junction, and amygdala (Denny et al., 2012; Lieberman et al., 2019; Coley et al., 2021; Simon et al., 2021). In the present study, mPFC showed increased FC with the right intracalcarine cortex in those scoring high in SPS+ traits, and increased FC with LOC that was positively correlated with both SPS trait clusters (indicating that individuals with higher overall levels of SPS tend to show stronger connectivity between these regions). The intracalcarine cortex houses the primary visual cortex, and the LOC is involved in higher-order visual functions including processing of objects (Lacey et al., 2009; MacLean et al., 2023) and integrating visual information about objects and actions (Ay and Demiralp, 2024). As such, the stronger FC we observed between mPFC and these regions associated with SPS may reflect a bottom-up approach to empathy, whereby information gathered from visual stimuli influences empathic processing, self-reflective processing, and decision-making. These results are consistent with findings from a magnetoencephalography study by Hamada et al. (2022) comparing brain activity of high empathizers versus low empathizers while participants watched films of social interactions. In this study, the high empathy group had greater activity in occipitotemporal regions (including LOC) than the low empathy group when they began viewing the social interaction films. The researchers inferred that participants with greater empathy paid more attention to the faces and bodies of characters in the films (Hamada et al., 2022). Because individuals with SPS display greater empathy, and because FC between neural regions can reflect task-based neural activity (Bressler and Menon, 2010), the stronger FC we observed between mPFC and these regions may relate to the fact that individuals with SPS pay more attention to visual stimuli (including interpersonal and contextual cues) and process these stimuli deeply to gain insight into others’ feelings (Aron and Aron, 1997; Aron et al., 2012).

A complementary explanation for the increased FC between the mPFC and both the LOC and left superior parietal lobule associated with features of SPS involves the trait’s link to mental imagery, which is considered to be a weak form of perception (Pearson et al., 2015). Jakobson et al. (2024) argued that individuals scoring high on SPS, who are fantasy prone and emotionally reactive, are more likely than less sensitive individuals to utilize imagery-based strategies to help them understand the thoughts and feelings of others, and to empathize with them. Both the LOC and superior parietal lobule are activated during tasks involving visual mental imagery (Lacey et al., 2009; Stokes et al., 2009; Liu et al., 2022). Therefore, the increased FC between the mPFC and these regions may reflect the frequent application of mental imagery during the deep processing that is thought to underpin heightened empathy in individuals scoring high on SPS traits.

### Heightened interoceptive and emotional processing and possible links to distress and emotion dysregulation

As previously described, personal distress was the main empathy-related construct sampled by the self-report empathy measure used in the current study that varied in its relationships to SPS+ and SPS− traits. Specifically, SPS+ was associated with experiencing low levels of personal distress, while SPS− was associated with experiencing high levels of personal distress. This suggests that previously described associations between SPS and both low tolerance to distress and emotion dysregulation (Brindle et al., 2015), may be largely accounted for by stronger expression of the SPS− trait cluster, specifically. We propose that heightened personal distress and emotion dysregulation experienced by individuals scoring high in SPS− traits when empathizing may explain, in part, a number of the rsMRI results found in the current study.

The insula is involved in many functions; of particular interest to the current study are its roles in interoception and emotion processing, which are vital to empathy (Singer et al., 2009; Uddin et al., 2017). Connectivity between the right insula and primary somatosensory cortex was associated with stronger expression of SPS− traits. A study by Straube and Miltner (2011) found increased brain activity in the right (posterior) insula and primary somatosensory cortex when participants were instructed to reflect on their own emotional experience while viewing threatening emotional pictures. Given this, the enhancement in FC we observed between these regions could suggest a neural mechanism that explains how sensitivity to unpleasant sensations in the body triggered by seeing others’ suffering may be translated into increased emotional reactivity and feelings of personal distress in those scoring high on SPS−.

The insula also works in conjunction with brainstem structures to integrate emotional and sensory information and autonomic responses through afferent and efferent connections (Sklerov et al., 2019). SPS− traits were associated with reduced FC between the left insula and the brainstem. This may contribute to ineffective integration of emotional, sensory, and autonomic information in individuals scoring high on SPS−, which could play into the ease with which these individuals become distressed and overwhelmed by unpleasant sensory and emotional stimuli.

Reduced FC between the left IFG pars triangularis and LOC was associated with SPS− traits. As previously described, the LOC is involved in higher-order visual processing (Lacey et al., 2009) and the left LOC, specifically, is activated during the processing of visual information related to emotion valence (Kuniecki et al., 2018). The left IFG, in contrast, is activated during emotion regulation, potentially due to the use of inner speech to label the experienced emotion (Morawetz et al., 2016). Given these findings, the decreased FC we found between the left IFG and LOC that was related to SPS− traits may reflect reduced ability to effectively regulate negative emotions triggered by visual stimuli. As previously noted, individuals scoring high on SPS− traits tend to use maladaptive forms of emotion regulation (Eşkisu et al., 2022). This may be especially true when they are experiencing empathic overarousal, potentially interfering with their ability to act with compassion. Emotion dysregulation may also exacerbate (Fernandes and Panwar, 2024) and/or be exacerbated by Brindle et al. (2015) other forms of negative affectivity (e.g., depression, anxiety, stress) that frequently impact those scoring high on SPS− traits (Attary and Ghazizadeh, 2021; Jakobson et al., 2024).

Stronger FC between the left IFG and right frontal pole was also correlated with strong expression of SPS− traits. This may relate to the fact that these regions are coactivated during episodic and working memory tasks (Bludau et al., 2014). When empathizing with others in difficult circumstances, we draw on episodic memory to assist with envisioning how they might be feeling (Gaesser and Schacter, 2014). Although this can evoke feelings of concern for the other person, it can also increase feelings of personal distress (Gregory et al., 2024).

We also observed stronger FC between the precuneus seed and the left frontal pole in those scoring higher on SPS− traits. The precuneus is a major node of the default mode network, which is involved not only in episodic memory, but also in self-reflective processing and rumination (Dadario and Sughrue, 2023). Those scoring high on SPS− traits may engage frequently in these latter processes, given their susceptibility to overarousal and feelings of personal distress. Indeed, Hoffman (2008) argues that experiencing empathic overarousal is likely to lead an individual to shift attention to their own distress, at the expense of engaging with the empathy target. Self-reflection and rumination may also lead one to catastrophize and overestimate the severity of one’s depression symptoms, which Kawakami et al. (2024) found to be linked to increased FC between the precuneus and frontal pole.

Finally, the relationship between SPS− traits and feelings of personal distress may also be partially explained by reduced FC between the precuneus and both the left occipital pole and the right cerebellum Crus I and lobule VI. These findings are interesting given that these alterations in FC might contribute to disrupted processing of emotionally-salient stimuli (Stoodley and Schmahmann, 2009, 2010; Halbertsma et al., 2020; Van Overwalle et al., 2020), potentially leading those scoring high in SPS− traits to feel overwhelmed and distressed in tense interpersonal situations. For example, lobule VI and Crus 1 in the cerebellum have been linked with both the processing of emotionally salient information and empathy (Stoodley and Schmahmann, 2009, 2010). The reduced FC between these regions and the precuneus may reflect impaired emotional processing, emphasizing feelings of distress in individuals with high SPS− traits.

### Limitations and future directions

Although the current study provides new information about the relationships between functional connectivity, SPS, and empathy, there are some limitations that must be addressed in future research. Participants were undergraduate psychology students, which limits the generalizability of results. In addition, although the current sample size was acceptable by current standards in neuroimaging research, it was not a large enough sample to find small effects. With a larger sample size, future researchers investigating the neural bases of SPS could incorporate additional covariates such as measures of alexithymia, emotion regulation, and/or negative affectivity. This may be informative, given the established links between SPS and these variables (Brindle et al., 2015; Attary and Ghazizadeh, 2021; Jakobson et al., 2024).

This research provides novel insights into the neural substrates underpinning the strong links that have been described between facets of SPS and empathy. We measured SPS+ and SPS− facets using averages of subscale scores across two SPS measures, as suggested in a comprehensive review on measuring SPS (Aron et al., 2012) and findings from a recent factor analysis (Jakobson et al., 2024). Recently, De Gucht et al. (2022) created a self-report measure of SPS that includes a broader range of SPS+ traits. Future research may benefit from utilizing this new measure in comparison to the combined SPS measures used in the current study.

Seven seeds were investigated in the current study, based on prior research implicating them in empathy and/or emotion processing. It was hypothesized that the SPS trait components would be associated with FC between these seeds and other brain regions, due to the strong links between SPS and empathy that have been reported. Interestingly, we saw no relationships between either SPS+ or SPS− trait scores and altered FC between three of these seeds (the anterior cingulate cortex, posterior cingulate cortex, and amygdala) and other brain areas. This was unexpected as Acevedo et al. (2021) had previously identified relationships between an overall measure of SPS and altered connectivity with these seeds. Given methodological differences between their study and the current investigation, and recent calls to use larger samples to increase the reliability of findings in fMRI studies (Marek et al., 2022), more work is needed to clarify how robust these associations are.

Finally, the results provide important new information that may shed light on why scoring high on SPS− traits may increase one’s vulnerability to the experience of personal distress while empathizing. In particular, strong expression of SPS− traits was linked to alterations in the FC between multiple neural regions that could contribute to emotion dysregulation. Future research should be directed at exploring why strong expression of SPS+ traits appears to offer some protection against becoming overwhelmed by these feelings.

## Data Availability

The raw data supporting the conclusions of this article will be made available by the authors, without undue reservation.

## Footnotes

1 “Deep” processing, as originally described by Craik and Lockhart (1972), refers to encoding and processing information in a meaningful and elaborate way that supports long-term memory. It may involve things such as the formation of mental images and/or the linking of current information to previous knowledge.

2
http://www.fil.ion.ucl.ac.uk/spm
